# Quantitative Systems Pharmacology: A Case for Disease Models

**DOI:** 10.1002/cpt.528

**Published:** 2016-11-18

**Authors:** CJ Musante, S Ramanujan, BJ Schmidt, OG Ghobrial, J Lu, AC Heatherington

**Affiliations:** ^1^ Pfizer IncCambridgeMassachusettsUSA; ^2^ GenentechSouth San FranciscoCaliforniaUSA; ^3^ Bristol‐Myers SquibbPrincetonNew JerseyUSA; ^4^ No institutional affiliation at the time of this research; ^5^ AstraZenecaCambridgeUK

## Abstract

Quantitative systems pharmacology (QSP) has emerged as an innovative approach in model‐informed drug discovery and development, supporting program decisions from exploratory research through late‐stage clinical trials. In this commentary, we discuss the unique value of disease‐scale “platform” QSP models that are amenable to reuse and repurposing to support diverse clinical decisions in ways distinct from other pharmacometrics strategies.

Significant progress has been made in the last 25 years integrating modeling and simulation approaches into the drug discovery and development process. Pharmacokinetic (PK) and pharmacodynamic (PD) models are broadly applied to address relevant questions at each stage of development. These models have helped inform drug discovery, development, and regulatory decisions and reduce the time and cost of bringing new drugs to market.^1^ Furthermore, successful application has set the stage for novel modeling approaches, including quantitative systems pharmacology (QSP).^2^

QSP has been defined as “the quantitative analysis of the dynamic interactions between drug(s) and a biological system that aims to understand the behaviour of the system as a whole…”^3^ It is precisely this focus on quantitative, mechanistic biology and pharmacology that has increased interest in QSP. Drug development is challenged by low success rates, with costly, late‐stage attrition primarily due to lack of efficacy.^4^ Earlier and more thorough testing of mechanisms of action of novel agents have been proposed as critical for reducing attrition. With its focus on the interplay of pharmacological and biological mechanisms, QSP is well poised to support this call.

This commentary stems from a workshop of the same name at the 2016 American Society for Clinical Pharmacology and Therapeutics (ASCPT) Annual Meeting. Discussion highlighted the need for communication of the benefits of QSP disease modeling approaches in clinical program decision‐making. Herein, we aim to:

- Discuss differences between fit‐for‐purpose and multi‐use disease platform QSP models;
- Demonstrate how QSP disease platforms can inform clinical programs in ways distinct from other PK/PD strategies;
- Illustrate current use of QSP disease platforms to guide clinical development;
- Guide the reader to resources for further understanding of QSP models and their application to clinical development.

## Disease‐scale QSP models

**Figure 1:**
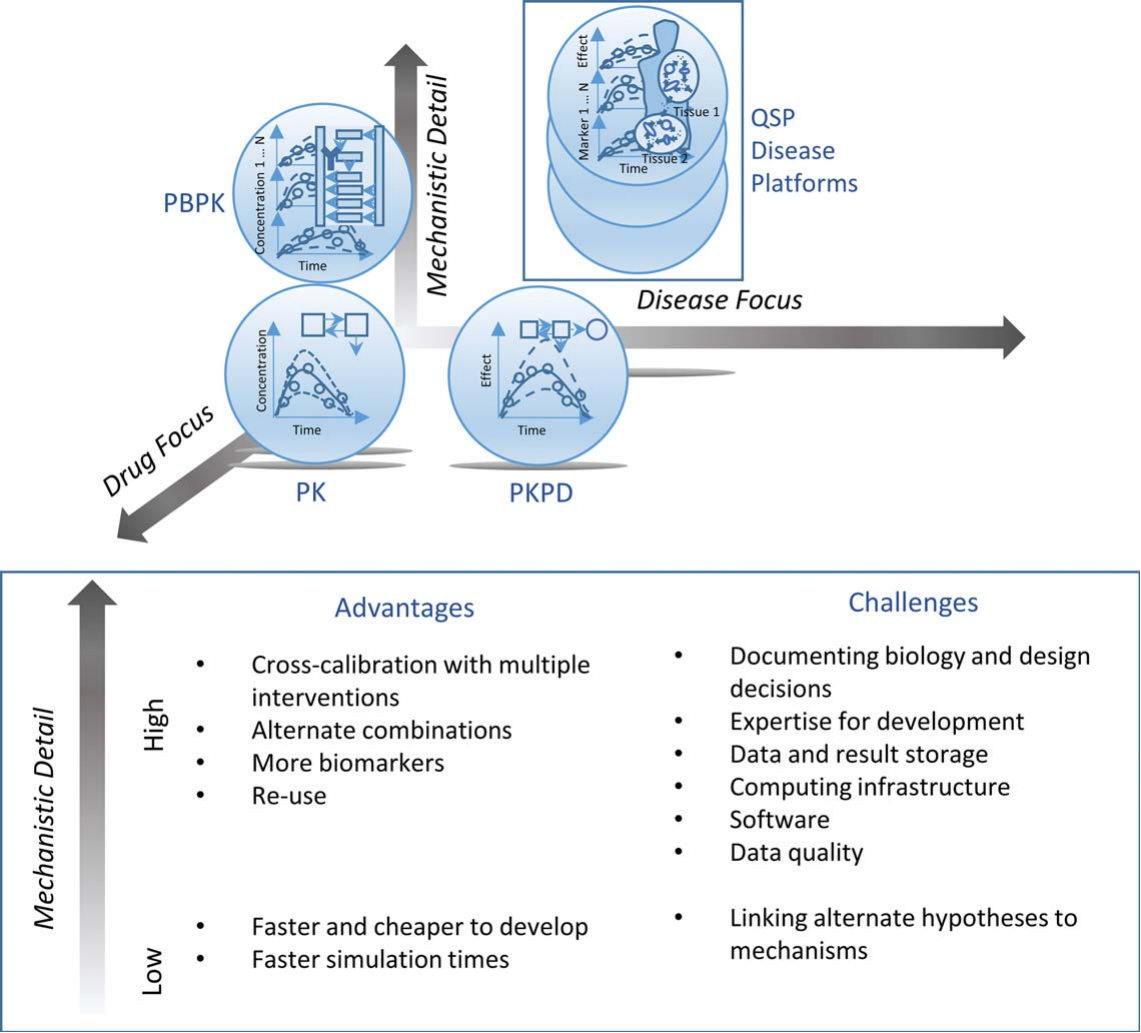
Schematic contrasting quantitative systems pharmacology (QSP) disease platform models and other modeling approaches used to support clinical pharmacology programs. Although empirical pharmacokinetic (PK) and PK/pharmacodynamic (PD) approaches are developed to adequately describe gathered data, they are generally not designed to make quantitative insights into specific underlying mechanisms and facilitate the use of these mechanistic insights to extrapolate to new conditions in which the relationship between model inputs and observed outputs might be qualitatively different. Mechanistic PK/PD can help address this, but only within the typically focused, minimal biology needed to relate existing target and output data. Approaches, such as pharmacologically based PK (PBPK) modeling, facilitate the use of additional mechanistic data to make predictions of drug disposition for new populations or when administering combinations of interacting drugs. Analogously, QSP disease platform models enable the use of mechanistic data for predictions of efficacy or changes in a safety signal. Similar to other modeling approaches, a continuum of potential model complexity exists depending on the variety of mechanisms and scale to which they will be mathematically characterized, and an appropriate approach should be identified based on the objectives.

Traditional empirical or mechanistic PK/PD models are designed to characterize one or more specific but similar datasets to generate inferences and predict results for related scenarios. PK/PD modeling emphasizes parsimony and parameter identifiability, and routinely incorporates population variability and uncertainty in parameter estimates. QSP models, meanwhile, are designed to investigate the effects of drug action on emergent behaviors of the underlying system, be that a pathway, cell, tissue, organ, or multi‐organ/whole‐body process (**Figure** 1). To do so, QSP models integrate datasets from diverse studies, contexts, and spatiotemporal scales into a mathematical framework that reflects our knowledge of the system. These models exploit this integrated, mechanistic representation to predict outcomes in untested scenarios, prioritizing relevant biological detail over identifiability. Explicit representation of mechanistic variability enables exploration of parameter uncertainty, population heterogeneity, and alternate biological hypotheses. However, due to their mechanistic detail and larger scale, QSP model development generally requires more time and biological knowledge than PK/PD approaches.

Within the broad category of QSP,^5^ disease‐scale platforms differ from fit‐for‐purpose models in that platforms typically: 1) include the interplay of multiple drug targets, pathways, and tissues in disease; 2) include a representation of the untreated disease state and a broad range of disease phenotypes; 3) mechanistically link target modulation to biomarker changes and outcomes; 4) allow comparisons against current standards of care; 5) support multiple applications, such as evaluation of the impact on efficacy and/or safety of different novel monotherapy and combination therapy treatments and alternate study designs (dose, regimen, and duration); and 6) can be reused, adapted, and repurposed for new treatments, questions, and indications.

## Application of disease‐scale QSP models to inform clinical programs

Two illustrative examples of disease‐scale QSP models, presented in the ASCPT workshop, highlight recent applications in clinical development.

## Lipoprotein metabolism and kinetic platform

Various measures of high‐density lipoprotein (HDL) are correlated with the risk of cardiovascular disease. Multiple drugs targeting the cholesteryl ester transfer protein (CETP) increased HDL‐cholesterol in the clinic and progressed to phase III trials. Yet, to date, they have failed to reduce cardiovascular risk, bringing into question whether HDL‐cholesterol is a good biomarker.

The lipoprotein metabolism and kinetic model was developed at Roche from diverse *in vitro* and human data.^6^ The model is a “mid‐sized” platform with over 24 state variables and parameters, developed and extended over a 1.5 year period. Its development and use proceeded in stages: (i) initial construction using publicly available data; (ii) adaptation and refinement using proprietary clinical data on a novel CETP inhibitor^7^; (iii) simulation and comparison of the effects of different targets and compounds on reverse cholesterol transport, the process of cholesterol removal from the periphery by HDL, which is believed to reduce cardiovascular risk.

Numerous targets were represented in the platform. Clinical data were collected from literature for calibration and validation to ensure the model recapitulated known effects of genetic mutations. All investigations were performed in a collection of “virtual patients,” or alternate parameterizations, representing biological variability and mechanistic uncertainty. The detailed representation of HDL metabolism enabled assessment of the impact of clinical dose regimen on the rate of reverse cholesterol transport, which is not directly measured in trials and, therefore, not amenable to PK/PD modeling.^7^

After a phase III trial revealed that the CETP inhibitor failed to reduce cardiovascular risk, the model was used to provide a mechanistic explanation for the results. It was then used to evaluate the potential use of another CETP inhibitor for a different indication. Subsequently, the model was applied to other programs and mechanisms targeting HDL. In contrast to CETP, modulation of other targets was predicted to increase the reverse cholesterol transport rate and, therefore, potentially decrease cardiovascular risk. To evaluate other clinical opportunities in HDL biology, the model was applied to compare simulated impact of a number of therapeutic interventions.^8^ The platform model provided a quantitative basis for differentiating the amount and time‐scale of potential plaque reduction with these interventions and contributed to strategic decisions on preclinical and clinical molecule development. The lipoprotein metabolism and kinetic example illustrates how a QSP platform was reused and extended to address diverse questions and inform multiple programs and decisions.

## Immuno‐oncology platform

With the US Food and Drug Administration approval of breakthrough therapies that block immune “checkpoints” to promote antitumor immunity and establish durable responses, the potential of immunotherapy is being realized. This raises fundamental questions of which checkpoints to target, how to optimize therapy timing and sequencing to take advantage of immune system dynamics, how therapeutic strategies should vary between indications, and how to best combine immunomodulatory mechanisms. Disease‐scale QSP platforms have previously addressed similar clinical development questions in order to mechanistically unravel, interpret, and guide optimal intervention in complex multifactorial autoimmune disorders.^5^, ^9^ To address these questions and support a diverse clinical pipeline in immuno‐oncology (I‐O), a mechanistic model of the cancer‐immunity cycle was developed. The platform includes representation of numerous immune cells, soluble factors, and cell surface interactions in the tumor, a sentinel lymph node, and blood.^10^ The initial focus for model development and application has been melanoma, in which clinical data are most abundant, with planned adaptation to other tumor types.

The development of the melanoma I‐O QSP platform has proceeded in two stages: an initial pilot project simulated the blood and tumor microenvironment and response to approved I‐O agents, ipilimumab and nivolumab; and a second stage in which the model was expanded to include a draining sentinel lymph node and additional cell types, soluble factors, cellular targets, and therapies. The first stage demonstrated reasonable dynamic trajectories in key biomarkers, including gamma interferon (IFN‐γ), CD8 T cells, CD4 regulatory T cells, and interleukin‐10, and suggested potential pathways limiting therapeutic response to target engagement with concomitant or sequenced clinical dosing strategies.^10^ With subsequent expansion, over 30 cell types, many with multiple activation states, over 30 soluble factors, and numerous regulatory interactions were included based on established roles in immunology, known or suspected importance for I‐O, or relevance to clinical development programs. The resulting model includes over 300 ordinary differential equations, has involved a 2‐year timeline, and has required a substantial continued investment of varied scientific, modeling, and computational expertise.

Currently, the melanoma I‐O QSP platform is being deployed to explore the efficacy of alternate ipilimumab and nivolumab therapeutic regimens. Additional planned applications include investigating the requirement for maintenance therapy and exploring combinations and sequencing with anti‐CD137, anti‐GITR, anti‐LAG3, and other therapies in clinical development at Bristol‐Myers Squibb.

## SUMMARY

QSP disease platform models provide an integrated, quantitative approach to clinical development, complementary and distinct from other PK/PD and pharmacometric approaches. The above examples, and others discussed in the literature,^1^, ^5^ demonstrate the potential for QSP disease platforms to provide continued value across a range of projects and disease indications. Notably, QSP recently has reached the regulatory arena with the use of an established bone metabolism platform in a US Food and Drug Administration Advisory Meeting Clinical Pharmacology Review.^5^

Although a QSP approach may involve higher upfront investments compared with traditional PK/PD models, QSP disease platforms enable mechanistic extrapolation between different contexts and facilitate reuse of quantitative knowledge from multiple development programs. Some key learnings from our examples are:

- A QSP disease model provides a common, integrated platform that serves as a predictive tool and an integrated quantitative knowledge repository for continued preclinical and clinical application;
- QSP models enable *in silico*, multivariate, and quantitative exploration of key mechanistic uncertainties and population variability^5^;
- Staged platform development, based on specific project needs, allows the resource investment to be spread out over time with staged application and return on investment.

For readers interested in learning more about QSP, in addition to references cited below, tutorials, reviews, and original research papers (along with technical detail allowing model reproduction) are available through the journal *CPT: Pharmacometrics & Systems Pharmacology*. However, there is a continued need for open‐source model dissemination to enable community and regulatory evaluation and use, and for public disclosure of the clinical impact of QSP platform models. These efforts will ultimately advance the broader adoption of this promising approach in clinical drug development.

## ACKNOWLEDGMENTS

The authors gratefully acknowledge Michael Zager and Jeffrey Woodhead for their assistance in conceptualizing the ASCPT workshop, and the International Society of Pharmacometrics QSP Special Interest Group and the ASCPT Systems Pharmacology Community for co‐sponsoring the session.

## CONFLICT OF INTEREST

The authors declared no conflict of interest.
